# Biochemical, Molecular and Behavioral Changes Induced by High-fat and High-sugar Diets: A Systematic Review of Non-clinical Studies

**DOI:** 10.1007/s12035-026-05816-w

**Published:** 2026-03-26

**Authors:** Camila Bach, Julia Vicentin Souza, Cláudia Sirlene Oliveira, Rosiane Guetter Mello, Daniele Maria-Ferreira

**Affiliations:** 1 Programa de Pós-Graduação Em Biotecnologia Aplicada À Saúde da Criança E Do Adolescente, Faculdades Pequeno Príncipe, Curitiba, 80250-200 Brazil; 2 Instituto de Pesquisa Pelé Pequeno Príncipe, Av. Silva Jardim No 1532, Curitiba, 80250-200 Brazil

**Keywords:** High-fat diet, High-sugar diet, High-calorie diet, Cognitive function, Cognitive decline

## Abstract

Diets that promote excessive caloric intake, particularly those high in fat and/or sugar, can cause harmful changes in the human body, including the brain. Excess consumption of fat and sugar may impair neuronal function and have both short- and long-term adverse effects. This systematic review examined the influence of high-fat and/or high-sugar dietary patterns on biochemical, molecular, and behavioral changes (PROSPERO protocol number: CRD42024526471). A systematic search was conducted in PubMed, EMBASE, and Scopus, and twenty-eight articles were included for data extraction. The studies indicate that obesogenic and energy-dense dietary patterns induce physiological changes in both the peripheral and central nervous systems, leading to neuronal, functional, and structural adaptations accompanied by biochemical, molecular, and behavioral changes. These adaptations appear to be associated with coordinated changes in gut microbiota composition, glucose and insulin metabolism, and bile acid signaling pathways. These findings support further investigation into strategies for the prevention and treatment of diet-related cognitive impairment. However, additional non-clinical and clinical studies are needed to better elucidate the mechanisms linking energy-dense dietary patterns and excessive caloric intake to these neural changes.

**Graphical Abstract:**

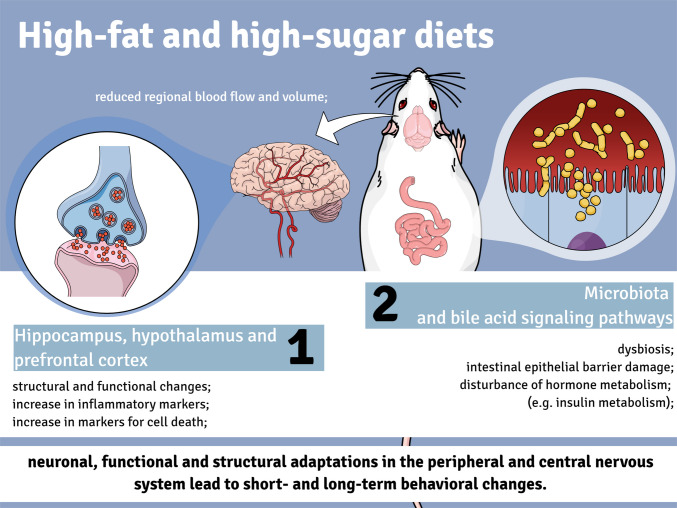

**Supplementary Information:**

The online version contains supplementary material available at 10.1007/s12035-026-05816-w.

## Introduction

The brain is the most complex organ of the human body and accounts for about 2% of the total body weight. It has a considerable and constant energy requirement and consumes about 20% of the total energy from nutrients [1–3]. An adequate intake of macro- and micronutrients is essential for efficient neuronal function, synaptic activity, and the synthesis of neurotransmitters and protein components [4, 5].

A growing body of neuroscientific evidence shows that adult mammal brains can undergo structural and functional remodeling in response to learning or experience [6, 7]. This process enables adaptive plasticity in mammalian brains, supporting learning, memory formation, and recovery of neural circuitry after injury, as demonstrated in experimental animal models [8]. The neurophysiological processes associated with these events range from rapid adjustments in synaptic strength, which are transient in nature, to long-term structural changes [9]. Important contributing factors are the formation of new synaptic connections, new neurons, new blood vessels, and non-neuronal glial cells in the brain [10]. The extent of neuronal remodeling that is triggered in response to learning or experience depends in part on factors that influence brain activity and the predisposition to trigger that activity. These factors include the quality of sleep, physical activity, and diet, which are important pillars of mental health due to their effects on the structure and function of the brain [11].

In fact, it has long been recognized that regular physical activity combined with adequate sleep and a good diet is essential for a healthy lifestyle and the prevention of various health problems [11]. Diet, in particular, is a recognized lifestyle factor that can influence brain structure and function and has the potential to alter predisposition to neuronal remodeling [4, 5, 12]. In 2018, a cross-sectional study showed that consumption of raw fruits and vegetables predicted a reduction in depressive symptoms and an increase in positive mood and life satisfaction [13]. In 2020, another cross-sectional study confirmed that the consumption of raw fruits and vegetables in addition to adequate sleep and physical activity improved depressive symptoms and well-being in young adults [11]. Conversely, recent evidence suggests that a diet high in sugar and saturated fat can significantly affect key elements of both neuronal and behavioral plasticity [14, 15], supporting the notion that dietary changes can have deleterious effects on the brain [16].

Despite the wealth of information found in the literature, the role of a high-fat and/or high-sugar diet on biochemical, molecular, and behavioral changes in non-clinical studies is still not fully understood and has not been systematically organized. Therefore, the aim of this review is to systematically present and critically evaluate recent advances in scientific literature regarding the effects of a high-fat and/or high-sugar diet on these parameters. This review provides a comprehensive discussion of the animal models used in these studies, as well as an in-depth analysis of the study designs, behavioral outcomes, and associated biochemical and molecular changes.

## Methods

The present study followed the Preferred Reporting Items for Systematic Reviews and Meta-Analysis (PRISMA) guideline for systematic reviews and meta-analysis [17] and its protocol was registered in the International Prospective Register of Systematic Reviews (PROSPERO), under the number CRD42024526471.

### Research Question

To formulate the guiding research question, the acronym PICOS [18] was used, where “P” denotes the problem, “I” the intervention, “C” the comparison, “O” the outcome, and “S” the study. The following strategy was created for this study: P: rodents exposed to different diets (high sugar and/or high fat),I: different diets (high sugar and/or high fat); C: animals fed standard rodent chow; O: alterations in biochemical, molecular, or behavioral markers; S: Animal studies.

### Search Strategy

A comprehensive literature search was carried out in the PubMed, EMBASE, and Scopus databases. The articles included in this review cover the period between 2010 and 2023. The search terms considered in this review included “neuroplasticity" and “diet”. The search approach was adapted for each database as needed.

### Inclusion and Exclusion Criteria

The search focused on original non-clinical studies related to the influence of diet on rodents’ biochemical and molecular alterations in the brain, as well as potential behavioral alterations. It was limited to one publication period (2010 to 2023) and articles published in English. Studies in experimental rodent models (rats and mice) exposed to different diet types (high-fat, high-fat/high-sugar, cafeteria, or high-sugar diets) were included. Only studies with primary (i.e., biochemical and/or molecular assays) and, if possible, secondary (e.g. behavior assays) results were included. Clinical studies, conferences, book chapters, reviews, and studies conducted before 2010 were not included. Studies with animals with comorbidities, with animal species other than rodents, in silico, and in vitro studies were not included.

### Study Selection and Data Extraction

After the database search, the studies were retrieved to Rayyan (https://www.rayyan.ai/) and duplicates were removed. Title and abstracts were independently selected regarding the eligibility criteria by two authors (CB and JVS). The evaluation of the full texts was performed independently by two authors (CB and JVS). The data (diet type, animal strain, study design, behavioral, biochemical and molecular alterations, and main conclusions) were collected independently by two authors (DM-F and CSO) and organized in an Excel spreadsheet. All discrepancies were resolved by senior authors (DM-F and RGM) at all stages.

### Assessment of the Risk of Bias

All studies included in this review were assessed for methodological quality using the RoB SYRCLE tool [19], considering the risk of bias (RoB) for animal studies. The assessment was conducted by two independent authors (CB and JVS) and included observation of the following characteristics, i) randomized housing, ii) description of allocation, iii) incomplete outcome data, iv) selective outcomes, and v) other sources of bias: statistical analysis and conflict of interest statement. For each analysis, there were three possible ratings (“low risk”, “high risk” or “uncertain risk”). If the information was unclear, the rating “no information” was selected. Articles with a high risk of “other sources of bias” were rated as high risk overall.

## Results

### Characteristics of the Systematically Selected Studies

**Fig. 1:**
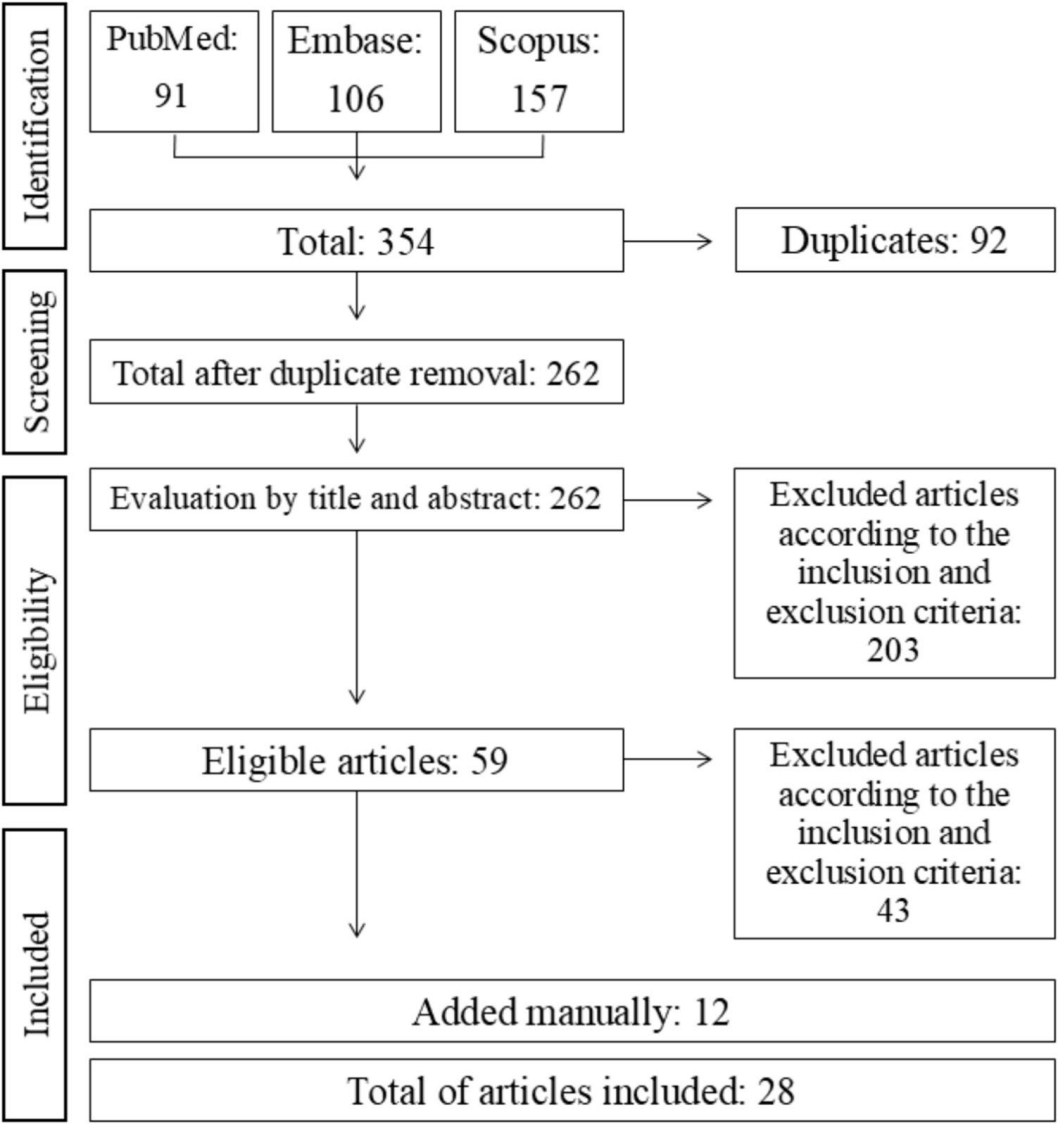
Flowchart of studies covered by the search strategy used to produce this review

Following a literature search, we identified 354 studies, and 92 duplicates were removed, totaling 262 studies for title and abstract review. After applying predefined inclusion and exclusion criteria, 59 studies examining the effects of various dietary interventions on biochemical, molecular, and behavioral outcomes were retrieved for full-text assessment. After detailed evaluation, 16 studies met all eligibility criteria, while 43 were excluded for not fully complying with the inclusion criteria upon full-text review. Additionally, 12 relevant articles were identified through manual searches of reference lists and complementary sources. Consequently, a total of 28 studies were included and qualitatively analyzed in this systematic review (Fig. 1). From those 28 studies, 11% evaluated the effects of a cafeteria diet, 22% examined the high-fat and high-sugar diet, 63% examined the effects of a high-fat diet and 7% assessed the effect of a high-sugar diet on biochemical, molecular, and/or behavioral alterations in rodents. It is worth noting that one study assessed more than one type of diet.

### Assessment of the Risk of Bias

**Fig. 2:**
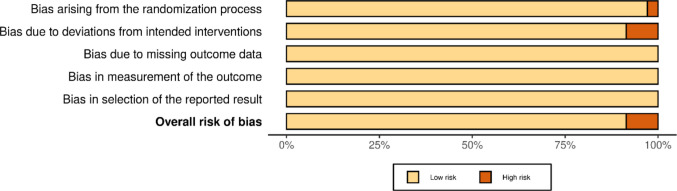
Assessment of methodological quality and risk of bias using the Rob SYRCLE tool for animal studies. The percentage of articles found in each category is shown in color and in bars, with green for low-risk studies, red for high-risk studies, and blue for studies that did not report the information sought

The results regarding the risk of bias are presented in Fig. 2. The vast majority of studies showed a low risk of bias.

### Main Findings

The main characteristics and findings of the included studies are described in Table 1. Most studies (63%) used a high-fat diet, to investigate the effects of dietary imbalance on biochemical, molecular, and behavioral changes. The diets offered to the animals during the experimental protocols varied considerably in composition across studies. When specified, diets were mainly composed of different fat sources (including saturated fat (e.g., lard) and polyunsaturated fats such as sunflower oil), carbohydrates (e.g., corn starch, sucrose, and fructose), protein sources (e.g., casein), fiber, and cholesterol. However, several studies did not provide detailed information on the specific type or source of macronutrients. In some experimental models, authors used commercially available human foods to induce a high-calorie intake, including white chocolate, orange-flavored soda, sausages, cookies (e.g., pizza-flavored crackers, chocolate chip cookies, strawberry-flavored cookies, Monte Carlo, Scotch Fingers, and Oreos), cakes (e.g., chocolate mud pie, jam roll, and lamingtons), processed protein products (e.g., party cakes, dim sims, and dog roll), or chow supplemented with bacon mortadella.

**Table 1:** Main characteristics and findings of the included studies

Diet	Strain/Age/N	Study Design	Main Conclusions	Reference
High-fat diet (HFD; D12331, 5.56 kcal/g): 58% fat, 25.5% carbohydrate, and 16.4% protein Standard control diet (CD; D12328; 4.07 kcal/g): 10.5% fat, 73.1% carbohydrate, and 16.4% protein	**Dams** C57BL/6N mice/4 weeks-old/~ 26 **Offspring** C57BL/6N mice/10 and 50 days-old/~ 208	F1 offspring were born to dams fed either a CD or an HFD for 70 days before mating and throughout pregnancy (~ 21 days). After weaning, all F1 offspring were fed standard rodent chow for approximately 50 days	Maternal consumption of an HFD disrupted neuroendocrine regulations in male offspring, while inflammatory-redox balance and emotionality are impaired in female offspring. Did not report the maternal or F1 offspring weight	[20]
High-fat diet (HFD; Research Diets Inc.): 60% kcal from fat Standard control diet (CD; Research Diets Inc.): 10% kcal from fat	**Dams** CD-1 IGS mice/N.I./~ 16 **Offspring** CD-1 IGS mice/21 and 112 days-old/~ 96	F1 offspring were born to dams fed either a CD or an HFD for 42 days before mating and throughout pregnancy and lactation (~ 42 days); after weaning, offspring either were analyzed immediately or were maintained on standard rodent chow for approximately 90 days	Maternal HFD intake causes anxiety- and depressive-like behaviors in F1 offspring, as well as alters the expression of genes involved in the neuroplasticity process. HFD caused a 20% body weight increase compared to CD in dams and F1 offspring	[21]
High-fat diet (HFD): 25.7% fat (4.5% palmitic acid, 1.99% stearic acid, 0.12% palmitoleic acid, 6.86% oleic acid, 2.58% linoleic acid, 0.25% α-linolenic acid, and 0.19% arachidonic acid), 19.5% protein, 41.3% carbohydrate, 3.5% fiber Standard control diet (CD): 5.3% fat [corn oil], 21.2% protein, 57.4% carbohydrate, and 4.6% fiber	**Dams** Sprague–Dawley rats/44 weeks-old/16 **Offspring** Sprague–Dawley rats/N.I./~ 128 (F1); ~ 176 (F2); ~ 464 rats (F3)	F1, F2, or F3 offspring were derived from dams fed either a CD or an HFD for approximately 50 days; offspring were subsequently maintained on standard rodent chow for ~ 50 days, except for a subgroup of F3 offspring from chow-fed dams that received an HFD for ~ 50 days	Central insulin resistance appears to be responsible for the transmission of maternal HFD-induced brain vulnerability and cognition impairment to the next generations, through the posttranslational modifications of glutamate receptors in the hippocampus. Maternal weight was not reported. Maternal HFD consumption did not alter F1, F2, and F3 offspring weight	[22]
High-fat diet (HFD): 60% kcal from fat, 21% kcal from carbohydrates, and 19% kcal from proteins Standard control diet (CD): 13% kcal from fat, 67% kcal from carbohydrates, and 20% kcal from proteins	Male C57BL/6 mice/4 weeks-old/42	Mice were assigned to groups based on diet type and duration: CD for 7 days, HFD for 7 days, CD for 14 days, HFD for 14 days, or HFD for 7 days followed by CD for 7 days	HFD reduces dendritic tree complexity of DCX + cells and this effect was correlated with reduced BDNF expression. HFD animals showed increased caloric intake; however, body weight did not differ from CD animals	[23]
High-fat diet (HFD; saturated fat): 9 kj/g (~ 47.6% kcal) from fat (39.2% lard, 45.1% monounsaturated fatty acids, and 11.2% sunflower oil), 6.9 kj/g from carbohydrates, and 3 kj/g from proteins High-fat diet (HFD; polyunsaturated fat): 8.5 kj/g (~ 47.2% kcal) from fat (10.1% lard, 22.8% monounsaturated fatty acids, and 58.3% sunflower oil), 6.9 kj/g from carbohydrates, and 3 kj/g from proteins Standard control diet (CD): 2.7 kj/g (~ 20.9% kcal) from fat, 7.2 kj/g from carbohydrates, and 3 kj/g from proteins	Male Sprague–Dawley rats/N.I./36	Animals were allocated to groups and fed for 13 days with either CD, HFD-saturated fatty acids, or HFD-polyunsaturated fatty acids	HFD-saturated fatty acids alters the hypothalamic marker of inflammation and the exploratory/memory behavior. Body weight did not differ among groups; however, fat-derived energy intake was higher in both HFD groups compared to CD	[24]
High-fat diet (HFD; TP23300): 60% from fat (lard/soybean oil mixture; 10:1), 20.6% carbohydrate, and 19.4% protein Standard control diet (CD; TP23303): 70.6% carbohydrate, 10% fat, and 19.4% protein	Male C57BL/6 mice/4 weeks-old/~ 46	Animals were assigned to groups and fed either CD or an HFD for 28 days	HFD intake effects in adolescence on emotion and neuroplasticity may be attributed to the microglial engulfment of nascent neurons. Body weight was not reported	[25]
High-fat diet (HFD; TP23300): 60% from fat (lard/soybean oil mixture; 10:1), 20.6% carbohydrate, and 19.4% protein Standard control diet (CD; TP23303): 70.6% carbohydrate, 10% fat, and 19.4% protein	Male C57BL/6 mice/4 weeks-old/~ 40	Animals were allocated to groups and fed either CD or an HFD for 35 days	HFD consumption induces microglial dysfunction and prefrontal neuroplasticity deficits associated with mood disorders in adolescent mice. HFD animals showed increased caloric intake and body weight compared to CD animals	[15]
High-fat diet (HFD): 60% from fat Standard control diet (CD): N.I	Male C57BL/6N mice/8 weeks-old/~ 20	Animals were allocated to groups and fed either CD or an HFD for 77 days	HFD consumption did not alter the markers of neuroplasticity in the amygdala. HFD animals showed increased body weight compared to CD animals	[26]
High-fat diet (HFD): 60% from fat Standard control diet (CD): N.I	Male C57BL/6 mice/8 weeks-old/N.I	Animals were allocated to groups and fed either CD or an HFD for 84 days	HFD intake disturbs the function of hippocampal astrocytes and induces depression-like behaviors, which could be related to alterations in glutamate transporters. HFD animals showed increased body weight compared to CD animals	[27]
High-fat diet (HFD): 60% from fat, 20% carbohydrate, and 20% protein Standard control diet (CD): 62.9% carbohydrate, 12.5% fat, and 24.6% protein	Male Sprague–Dawley rats/7 weeks-old/~ 75	Animals were allocated to groups and fed either CD or an HFD for 112 days	HFD intake causes alteration in endoplasmic reticulum stress proteins and apoptosis, which could be related to the neuroplasticity alterations. HFD animals showed increased body weight compared to CD animals	[28]
High-fat diet (HFD): 60% from fat (lard) Standard control diet (CD): 10% from fat	Male C57BL/6 mice/36 weeks-old/32	Animals were allocated to groups and fed either CD or an HFD for 140 days	HFD intake impairs immature neurons in the hippocampus. HFD animals showed increased body weight compared to CD animals	[29]
High-fat diet (HFD): 60% from fat Standard control diet (CD): N.I	Male C57BL/6 J mice/N.I./60	Animals were allocated to groups and fed either CD or an HFD for 140 days	HFD intake reduces PPAR levels and could be related to neuroinflammation with secondary degeneration of dopaminergic neurons. HFD animals showed increased body weight compared to CD animals	[30]
High-fat diet (HFD): 60% from fat Standard control diet (CD): 10% from fat	Male C57BL/6 mice/4 and 8 weeks-old/~ 50	Animals were allocated to groups and fed either CD or an HFD for 168 days	HFD intake effects on neurobehavior and hippocampal neuroplasticity were more pronounced in the young animals. HFD animals showed increased body weight compared to CD animals	[31]
High-fat diet (HFD, p1400f): 45 to 60% from fat Standard control diet (CD): N.I	Male C57BL/6 mice/N.I./N.I	Animals were allocated to groups and fed either CD or an HFD for 210 days	HFD intake alters hippocampal neuroplasticity via GLP-1R/BDNF/TrkB signaling. Body weight was not reported	[32]
High-fat diet (HFD): 60% from fat Standard control diet (CD): 10% from fat	Male C57BL/6 mice/4 and 8 weeks-old/~ 50	Animals were allocated to groups and fed either CD or an HFD for 252 days	HFD intake effects on neurobehavior and hippocampal neuroplasticity were more pronounced in the young animals. HFD animals showed increased body weight compared to CD animals	[33]
High-fat diet (HFD; TP23300): 60% from fat (lard/soybean oil mixture; 10:1), 20.6% carbohydrate, and 19.4% protein Standard control diet (CD; TP23303): 70.6% carbohydrate, 10% fat, and 19.4% protein	Male C57BL/6 mice/8 weeks-old/48	Animals were allocated to groups and fed either CD or an HFD for 168 or 252 days	HFD consumption alteration on behavior and hippocampal neuroplasticity appears to be attributable to alteration of microglial status, which is closely associated with the cellular lipid content. HFD animals showed increased body weight compared to CD animals	[34]
High-fat diet (HFD): 60% from fat (lard) Standard control diet (CD): N.I	Male C57BL/6 mice/~ 9 weeks-old/30	Animals were allocated to groups and fed either CD or an HFD for 504 days	HFD intake alters IGF-IRβ/PI3K/Akt/Gsk-3β and mTOR signaling pathways. HFD animals showed increased body weight compared to CD animals	[35]
High-fat high-sugar diet (HFHS): 20% fat (lard), 39.6% sucrose, and 19.4% protein Standard control diet (CD): N.I	Male Sprague–Dawley rats/~ 4 weeks-old/32	Animals were assigned to groups and fed either CD for 28 days or CD for 28 days with additional access to a HFHS diet for 2 h/day	HFHS diet intake caused physiological changes to the brain, particularly expression of mRNA associated with reward and neuroplasticity, which could explain the behavioral alterations. HFHS animals showed increased caloric intake and body weight compared to CD animals	[36]
High-fat high-sugar diet (HFHS): 20% fat (lard), 39.6% sucrose, and 19.4% protein Standard control diet (CD): 65% carbohydrates, 12% fat, and 23% protein	Male Sprague–Dawley rats/~ 4 weeks-old/24	Animals were assigned to groups and fed either CD for 28 days or CD for 28 days with additional access to a HFHS diet for 2 h/day	HFHS intake induces prefrontal cortex dysfunction and subsequent behavioral deficits. Neither body weight nor energy intake differed among the animals	[37]
High-fat high-sugar diet (HFHS): 46% fructose and 20% lard Standard control diet (CD): 5% cellulose, 20% casein, 25% maize starch, 25% potato starch, 16% maltodextrin, and 4% soybean oil	Male Wistar rats/8 weeks-old/60	Animals were allocated to groups and fed either CD or an HFHS for 84 days	HFHS intake altered several inflammatory and neuroplasticity markers. Neither body weight nor fat weight differed among the animals	[38]
High-fat high-sugar diet (HFHS): 29% fat, 34% sucrose, and 1.25% cholesterol combined with glucose/fructose-supplemented drinking water (42 g/L; 55%/45% w/w) Standard control diet (CD): 6.2% fat, 44% carbohydrate, and 18% protein	Male C57BL/6 mice/3 weeks-old/~ 10	Animals were allocated to groups and fed either CD or an HFHS for 203 days	HFHS intake caused neuroplasticity alteration through IL-17 signaling. Body weight was not reported	[39]
High-fat high-sugar diet (HFHS): 21.2% fat, 34% sucrose, and 0.2% cholesterol Standard control diet (CD): 5.2% fat, 12% sucrose, and 0.01% cholesterol	C57BL/6 mice/3 weeks-old/~ 12	Animals were allocated to groups and fed either CD or an HFHS for 252 days	HFHS diet intake disturbed the bile acid signaling pathways, which seems to be related to neuroplasticity impairment. Body weight was not reported	[40]
High-fat high-sugar diet (HFHS): 21.2% fat, 34% sucrose, and 0.2% cholesterol Standard control diet (CD): 5.2% fat, 12% sucrose, and 0.01% cholesterol	Male C57BL/6 mice/3 weeks-old/~ 12	Animals were allocated to groups and fed either CD or an HFHS for 287 days	HFHS diet intake disturbed the bile acid signaling pathways, which seems to be related to neuroplasticity impairment. HFHS animals showed increased body weight compared to CD animals	[41]
Cafeteria diet (CAF): CD supplemented with a variety of palatable human foods, including cakes, biscuits, and savoury protein-rich items (e.g., party pies, dim sims, dog roll), and 10% sucrose solution in addition to water Standard control diet (CD): N.I	Male Sprague–Dawley rats/N.I./20	Animals were allocated to groups and fed either CD or a CAF for 25 days	CAF diet intake disturbs inflammatory and neuroplasticity pathways. CAF diet increased caloric intake and body weight compared to CD	[42]
Cafeteria diet (CAF): 32% from fat, 57% from carbohydrates, and 10% from protein; CD supplemented daily with a variety of palatable high-fat, high-sugar human foods (e.g., meat pies, cakes, biscuits) Standard control diet (CD): 12% from fat, 23% protein, and 65% carbohydrate	Male Sprague–Dawley rats/6 weeks-old/32	Animals were allocated to groups and fed either CD or a CAF for 56 days	CAF diet intake altered hippocampal plasticity and caused contextual fear memory deficits. CAF diet increased body weight compared to CD	[43]
Cafeteria diet (CAF): 42% carbohydrates, 16% protein, and 42% lipids; CD supplemented ultra-processed, highly palatable foods (e.g., bacon mortadella, sweet biscuits, chocolate biscuits, pizza-flavored crackers, white chocolate, sausages, and sugar-sweetened soda) Standard control diet (CD): 63% carbohydrates, 26% protein, and 11% lipids	Male Wistar rats Age = 12 weeks-old N = 14	Animals were allocated to groups and fed either CD or a CAF for 140 days	CAF diet intake altered the marker of neuroplasticity. Body weight was not reported	[44]
High-sugar diet (HS): 2.69 kj/g from fat, 4.12 kj/g from complex carbohydrates, 4.2 kj/g from simple carbohydrates (sucrose), and 2.98 kj/g from proteins Standard control diet (CD): 2.7 kj/g from fat, 5.11 kj/g from complex carbohydrates, 2.05 kj/g from simple carbohydrates, and 3 kj/g from proteins	Male Sprague–Dawley rats/N.I./24	Animals were allocated to groups and fed either CD or an HS for 13 days	HS consumption did not alter the expression of the marker of neuroplasticity evaluated, but altered the hypothalamic marker of inflammation and the exploratory/memory behaviors. Body weight did not differ among groups; however, sucrose-derived energy intake was higher in both HS group compared to CD	[24]
High-sugar diet (HS): 1.5 g/100 g from fat (sunflower oil), 20.4 g/100 g from carbohydrates (fructose), and 9.2 g/100 g from proteins (casein) Standard control diet (CD): 1.5 g/100 g from fat (sunflower oil), 20.4 g/100 g from carbohydrates (cornstarch), and 9.2 g/100 g from proteins (casein)	Male Wistar rats Age = ~ 3 weeks-old N = ~ 32	Animals were allocated into three groups and fed either CD for 42 days, HS for 21 days, or HS for 21 days followed by CD for an additional 21 days	Several of the alterations in the frontal córtex (BDNF, CML, CEL, acetylcholinesterase activity, dysregulation of neurotransmitter levels) persisted after switching to the control diet, thus pointing to adolescence as a critical phase. HS did not alter body weight nor intake energy compared to CD	[45]

The time during which the animals were exposed to the diets ranged from a few days (e.g., 7 days) to weeks, months, or even years (e.g., 504 days). Mice or rats, male or female animals of different ages (from 3 to 64 weeks old) were used. In addition, some studies investigated whether the offspring of mothers exposed to a high-fat and/or high-sugar diet showed behavioral or neuronal changes. The animal strains used also varied widely. The studies included C57BL/6 and C57BL/6N mice, CD-1 IGS mice, and Sprague–Dawley and Wistar rats.

The behavioral changes induced by a high-calorie diet, as reported in the systematically selected studies, are presented in Table 2. While a few studies found no changes in locomotor activity and memory, most studies indicated that a high-calorie diet can cause various cognitive and other changes. Almost all studies described anxiety- and depression-like behaviors in both young and adult, male and female animals following acute or chronic exposure to high-calorie diets. Other effects included decreased exploratory behavior, social interaction and social motivation and recognition; decreased learning and memory and decreased cognitive flexibility; increased passive stress coping; cognitive impairment, and trace fear conditioning. In addition, the offspring of mothers exposed to a high-fat and/or high-sugar diet exhibited anxious and/or depression-like behaviors. The mothers also showed increased passive stress coping, spent more time away from the nest, and were less likely to nest-build.

**Table 2:** Behavioral changes induced by a high-calorie diet, as reported in the systematically selected studies

Diet	Type of Alteration	Alteration	Reference
HFD	Behavioral	↓ exploratory behavior in female offspring ↑ active coping strategy in female and male offspring	[20]
HFD	Behavioral and Social	**Dams** ↑ time spent away from the nest ↓ the frequency of nest building ↑ passive stress coping **F1 offspring** ↑ anxiety- and depressive-like behaviors ↑ passive stress coping ↓ social interaction	[21]
HFD	Behavioral and Cognitive	↓ learning and memory in F1, F2, and F3 ↓ learning and memory in F3 offspring fed with an HFD	[22]
HFD	Behavioral	↓ exploratory activity	[24]
HFD	Behavioral	↑ anxiety- and depressive-like behaviors	[25]
HFD	Behavioral	↑ anxiety- and depressive-like behaviors	[15]
HFD	Behavioral	↓ locomotor activity ↑ depression-like behaviors	[27]
HFD	Behavioral and Cognitive	↑ cognitive Impairment and anxiety-like behavior ↓ locomotor activity	[30]
HFD	Behavioral and Cognitive	↓ cognitive flexibility ↑ anxiety and depression-like behavior	[31]
HFD	Behavioral	↑ depression-like behavior	[32]
HFD	Behavioral	↑ anxiety and depression-like behavior	[33]
HFD	Behavioral	↑ anxiety and depression-like behavior	[34]
HFD	Cognitive	↓ spatial memory and learning	[35]
HFHS	Social	↓ social motivation and recognition	[46]
HFHS	Social	↓ social recognition	[37]
HFHS	Behavioral	↑ distance traveled, and time spent in the center	[39]
CAF	N/A	No alterations were observed in the memory and locomotor activities	[47]
CAF	Behavioral	↓ trace fear conditioning	[43]
HS	Behavioral	↓ exploratory activity	[24]

*HFD* high-fat diet, *HFHS* high-fat high-sugar diet, *CAF* cafeteria diet, *HS* high-sugar diet

Data was extracted from all eligible studies. For clarity and synthesis, Table 2 highlights behavioral outcomes with reported diet-related alterations. Behavioral tests with no significant differences between groups were not individually included in the table.

Table 3 shows the main biochemical and molecular alterations caused by a high-calorie diet, as reported in the systematically selected studies. In terms of biochemical and molecular changes, studies have investigated a variety of markers that indicate acute and chronic changes in neuronal remodeling and consequently confirm the above findings on cognitive decline and behavior. The hippocampus was the most frequently examined structure in the studies included in this systematic review. Changes described include increases and decreases in the expression of various molecules such as BDNF and inflammatory markers, including TNF-α, IL-1β, and IL-6. Changes were also described in the hypothalamus, prefrontal cortex, *substantia nigra*, striatum, and microglia. Some authors did not isolate specific structures for dosages but also showed changes in the general structure of the brain, and others provided results on plasma changes, such as changes in corticosterone levels.

**Table 3:** Biochemical and molecular alterations caused by a high-calorie diet, as reported in the systematically selected studies

Diet	Tissue/Organ	Biochemical and Molecular Changes	Reference
HFD	Plasma; Hippocampus	↓ basal plasma corticosterone levels in male offspring ↓ BDNF, NRF-2, and Keap-1 expression in the hippocampus only in the female offspring ↑ CD68 expression in male and female offspring ↑ Ucp-2 expression in female offspring	[20]
HFD	Whole brain; Prefrontal cortex	**Dams** ↑ brain TLR4 **Pups** ↑ prefrontal cortex GLUN2C and ZIF-268 expression in 21-day-old female offspring ↓ prefrontal cortex SYP expression 112-days-old offspring	[21]
HFD	Hippocampus	↓ *cornu ammonis* CA 3-CA1 hippocampal long-term potentiation (LTP) in the F1, F2, and F3 ↓ CA 3-CA1 hippocampal LTP in the F3 offspring fed with a HFD ↓ hippocampal CA1 dendritic spine density in F3 offspring and F3 offspring fed with an HFD	[22]
HFD	Hippocampus	↓ dendritic branches and dendritic intersections in the DCX-positive neurons in the hippocampus ↓ BDNF in the dorsal hippocampus	[23]
HFD	Hypothalamus	↓ hypothalamic NfkBia expression	[24]
HFD	Hippocampus	↓ Ki67- and DCX- positive cells in the hippocampus ↓ dendritic branches, dendritic length, and the number of dendritic intersections in the DCX-positive neurons in the hippocampus ↑ hippocampal Ibal- and CD68-positive cells ↑ microglial engulfment of DCX positive material in the hippocampus	[25]
HFD	Prefrontal cortex	↓ dendritic branches, dendritic length, and the number of dendritic intersections in the medial prefrontal cortex ↓ PSD95 density in the medial prefrontal cortex ↑ microglial phagocytosis of the PSD95 in the medial prefrontal cortex	[15]
HFD	Amygdala	No alteration in TrkB, BDNF, SYT-1, SYT-4, and SNAP-25 protein levels in the amygdala	[26]
HFD	Hippocampus	↑ activation of astrocytes (GFAP protein) in the hippocampus ↓ intersections and branches in GFAP-positive astrocytes in the hippocampus ↓ astrocytic neuroplasticity-related proteins (GLAST, GLT-1, and Cx-43) in the hippocampus	[27]
HFD	Prefrontal cortex	↑ FATP1, GRP78, p-PERK/PERK, p-elF2α/elF2α, caspase 12, CHOP, and Bax/Bcl-2 in the prefrontal cortex ↓ BDNF and SYN in the prefrontal cortex	[28]
HFD	Hippocampus	↓ dendritic branches in the DCX-positive neurons in the hippocampus	[48]
HFD	Nigrostriatal system (Substantia nigra + Striatum); Mesolimbic system	↓ dopaminergic neurons in the substantia nigra ↓ dendritic spine density in the substantia nigra and striatum ↑ Iba-1 and GFAP in the substantia nigra and striatum ↓ PPAR in the dopaminergic neurons of the substantia nigra and ventral tegmental area	[30]
HFD	Hippocampus	↓ dendritic branching, branch length, complexity of neuronal dendritic trees, and spine density in the hippocampus of 4 weeks initial age of treatment ↑ CD68-positive cells in the hippocampus of both ages evaluated ↑ microglial engulfment of PSD95^+^ puncta at 4 weeks after the initial age of treatment ↑ p-IR in hippocampal microglia 4 weeks after the initial age of treatment	[31]
HFD	Serum; Hippocampus	↓ serum 5-HT ↑ serum corticosterone, IL-1β, and IL-6 ↓ hippocampal BDNF, p-ERK/ERK, p-TrkB, p-AMPK, p-CREB/CREB, and GLP-1R	[32]
HFD	Hippocampus	↑ hippocampal insulin ↓ hippocampal pY-IR/IR in 4 weeks initial age of treatment ↓ hippocampal DCX positive cells in 4 weeks initial age of treatment ↓ hippocampal DCX and BDNF levels in 4 weeks initial age of treatment	[33]
HFD	Hippocampus	↓ hippocampal dendritic length, dendritic intersections, and dendritic spine density ↑ hippocampal CD68-positive cells ↑ microglia engulfment of PSD95 and lipid droplets in the hippocampus	[34]
HFD	Hippocampus	↑ hippocampal TNF-α, COX-2, Caspase-3, Iba-1, GFAP, p-JNK, AKT, mTOR, p-Tau, Beta-Amyloid ↓ hippocampal NeuN, Ki-67, p-CREB, BDNF, IGF-1R, JNK, PI3K, p-PI3K, p-AKT, GSK3β, p-mTOR, GLUT4, Tau, SAP 102	[35]
HFHS	Hippocampus; Prefrontal cortex	↓ BDNF, MaoA, COMT IL-6, NLRP3 expression in the prefrontal cortex ↓ MaoA expression in the hippocampus	[46]
HFHS	Prefrontal cortex	↓ the total number of parvalbumin-positive interneurons and perineuronal nets in the medial prefrontal cortex ↑ FosB/ΔFosB in the prefrontal cortex	[37]
HFHS	Serum; Hypothalamus; Hippocampus	↑ plasmatic IL-6 concentration ↓ hypothalamic IL-1R1, mineralocorticoid receptors, corticotropin-releasing hormone, NGF, and SYP expression ↑ hippocampal IL-1ra, glucocorticoid receptor expression	[38]
HFHS	Hippocampus; Whole brain homogenate/isolated microglia	↓ long-term potentiation (LTP) at Schaffer collateral–CA1 synapses ↓ BDNF and PSD-95 protein in the brain ↑ RoRyt, IL-17A, IL-22, IL-1β, IL-6, TNF-α, MCP-1, CCL-5, CCL-17, CCL-20, NOS-2, F4/80, CX3CR1, KCA3.1, KV1.3 expression in the brain	[39]
HFHS	Hippocampus; Frontal cortex; Whole brain	↓ long-term potentiation (LTP) at Schaffer collateral–CA1 synapses ↑ hippocampal IL-1β, IL-6, NOS2, CH25H, SAA1, TNF-α, CCL5, CCL17, CCL20, ApoE, KCA3.1, KV1.3, and KIR2.1 expression ↑ CD11b, pERK1/2, IL-6, IL-1beta, and TNF-α levels in the brain ↓ GPBAR1, BDNF, and PSD-9 levels in the brain ↓ BDNF, GPBAR1, PC1/3, NOS1, DIO2, GLP1R, PYY, FXR, CYP46A1, NOS3, SHP, CYP39A1, and CYP27A1 expression in the brain	[40]
HFHS	Hippocampus; Frontal cortex and ventral posteromedial thalamic nucleus; Whole brain homogenate/isolated microglia	↓ long-term potentiation (LTP) at Schaffer collateral–CA1 synapses ↑ IL-1β, IL-6, TNF-α, FOXp3, SAA1, CCL17, CCL20, ICAM1, KCA3.1, and KV1.3 expression in the brain and isolated microglia ↓ claudin1 expression in the brain ↓ Occludin, RARβ, CYP26A1, and ALDH1A1 expression in isolated microglia ↓ TGR5, GLP1R, PYY, FGF21, and BDNF expression in the brain and isolated microglia ↑ DCA and TCA levels in the brain ↓ βMCA, CDCA, UDCA, TLCA, and T-α-β-MCA levels in the brain ↑ HMGCR, SQLE, CH25H, CYP39A1, CYP7A1 expression in the brain ↓ CYP27A1, CYP7B1, CYP46A1, FXR, SHP expression in the brain ↑ CH25H, CYP7A1 expression in isolated microglia ↓ HMGCR, SQLE, CYP27A1, CYP7B1, CYP46A1, CYP39A1, FXR, SHP expression in isolated microglia	[41]
CAF	Hippocampus; Perirhinal cortex	↑ Homer1a, IGF1, Syn1, BDNF, CREB1, mTOR, GFAP, Ptgs2, 5HT1A, mGLUR5, Grin2b, MAPK8, MAPK10, Nr3c1, GLUT3, JAK2, and NOD2 expression in the hippocampus ↓ IKBKB and 5HT2c expression in the hippocampus ↑ BDNF expression in the perirhinal cortex ↓ MCP-1 expression in the perirhinal cortex	[47]
CAF	Hippocampus	↓ hippocampal Reelin expression	[43]
CAF	Hippocampus	↓ hippocampal synaptophysin levels	[44]
HS	Hypothalamus	↓ hypothalamic NfkBia expression	[24]
HS	Frontal Cortex	↑ Glut-5 and GFAP expression in HS feedings rats ↑ fructose, uric acid, TNF-α, Hpt, N-Tyr, TrkA, and acetylcholine levels in HS feedings rats ↓ Trkβ, dopamine, tyrosine, and tyramine levels in HS feedings rats ↓ Synaptophysin, synaptotagmin I, and PSD-95 expression in HS feedings rats ↑ AChE and MAO activity in HS feedings rats ↑ CML and CEL levels in HS and HS + rodent chow recover feeding rats ↑ pErk1/Erk1 and pErk2/Erka ratio in HS and HS + rodent chow recover feeding rats ↓ BDNF and pCREB levels in HS and HS + rodent chow recover feeding rats ↑ glutamate and GABA levels in HS + rodent chow recover feeding rats	[45]

*HFD* high-fat diet, *HFHS* high-fat high-sugar diet, *CAF* cafeteria diet, *HS* high-sugar diet

## Discussion

The concept of a healthy diet has evolved significantly in recent years, shifting from a primary focus on caloric adequacy and macronutrient balance to a broader emphasis on dietary quality and overall eating patterns. Epidemiological and non-clinical studies have shown that adequate nutrition, combined with physical activity and other lifestyle factors, plays a central role in promoting general health and well-being [49]. At the same time, research across multiple disciplines has consistently found that high consumption of Western-style, energy-dense, and ultra-processed foods is strongly associated with overweight, obesity, and an increased risk of metabolic and cardiovascular diseases, including diabetes [50].

The detrimental effects of consuming foods high in saturated fat, sodium, sugar, and easily assimilated carbohydrates appear to be due not only to their very low nutritional value but also to their tendency to promote non-homeostatic behaviors characterized by repetitive and excessive consumption of foods, which can alter gut-brain communication and the links between taste perception and nutrient response [51, 52]. If these changes persist, the previously established behavior can cause several important problems, such as a change in the homeostatic environment of the gut, intestinal inflammation, and hormonal problems that contribute to neuronal adaptations in the central nervous system, leading to more serious issues, including cognitive decline [53–55].

In fact, in this systematic review, we have described some studies that confirm the above. Most studies that have examined the negative effects of a high-fat, high-sugar diet or a cafeteria diet have primarily documented decreased performance on tasks that depend on good hippocampal and prefrontal cortex function, and interestingly, these findings have often been associated with cognitive decline, anxiety, and depression-like states. However, the mechanisms behind all the changes described are only partially understood. Some authors speculate that a high-calorie diet could affect the brain directly, while others put forward the idea that this may occur through an indirect effect via the periphery [54, 56–58].

A high-fat diet can alter cerebral vascular homeostasis by damaging the endothelium of cerebral microvessels, impairing the absorption of nutrients and the excretion of toxic substances, and leading to an increase in inflammatory markers in the brain [59]. A persistent state of inflammation in certain brain regions, including the hippocampus, hypothalamus, and prefrontal cortex, contributes to acute and chronic structural and functional changes and can lead to decreased regional blood flow and volume, activation of microglia, increased gene expression of proinflammatory brain mediators, including IL-1β and TNF-α [60], and increased markers of cell death [60–62]. Ultimately, it can lead to a reduction in specific biomarkers such as BDNF [14, 63, 64] and result in acute and long-term changes associated with significant alterations in brain reward circuitry, contributing to the development of anxiety and depression-like behaviors in animal models [65, 66]. Metabolic abnormalities such as decreased expression of glucose transporters and alterations in insulin metabolism can also cause neuronal changes at the morphological and physiological level, reflected in a reduction in short- and long-term potentiation response and a decrease in specific markers of synaptic neuronal plasticity [59, 67]. In addition, excessive consumption of certain nutrients, such as salt, can disrupt peripheral hormone metabolism, impair bile acid signaling pathways, alter the composition of the gut microbiota, and disrupt the protective barrier of the intestinal epithelium [67, 68]. These peripheral changes appear to exacerbate the negative effects of a high-calorie diet and influence the development of central nervous system problems via the gut-brain axis [39, 41].

Interestingly, some authors have investigated whether different times and different moments of exposure to a high-calorie diet can cause changes in the brain and behavior; or even whether exposure in utero can lead to altered phenotypes in the offspring and the risk of long-term morbidity. Long time spans [23, 26, 27, 69], but also very short periods of high-fat diets or cafeteria diets can impair cognitive function and inflammatory and neuroplasticity pathways in the brain [30, 35, 70]. Mechanistically, these early cognitive impairments appear to be driven by converging inflammatory, vascular, and neuroplasticity-related pathways triggered by exposure to high-fat diets**.** Using a high-fat diet providing 60% of total energy from fat, Kao et al. [30] demonstrate that HFD-induced neuroinflammation in nigrostriatal and mesolimbic circuits, marked by microglial and astroglial activation, reduced PPAR signaling, dopaminergic neuron loss, and decreased dendritic spine density is associated with cognitive deficits and anxiety-like behavior. Similarly, De Paiva et al. [35] show that an HFD (60% fat derived from lard) is sufficient to induce hippocampal inflammation, activation of stress and neurodegenerative pathways, and suppression of neurotrophic and synaptic plasticity signaling, culminating in impaired learning and memory. Extending these findings beyond diet exposure alone, Feng et al. [70] identify obesity-induced blood–brain barrier dysfunction as a critical upstream mechanism that facilitates peripheral inflammatory signaling into the brain and amplifies neuroinflammation and cognitive decline.

While some studies indicate that the effects are more pronounced in young animals [15, 25, 31, 33], others describe that the adverse effects do not depend on age and can extend over generations [34]. Offspring of mothers exposed to a high-fat and/or high-sugar diet also show significant changes, including metabolic syndrome, cardiovascular and cerebrovascular problems, and behavioral changes [20–22].

Recent research has also suggested that overconsumption of hyperpalatable diets could be associated with the development of low-grade inflammation [71]. Although research on this topic is still limited, preliminary evidence suggests that overconsumption of such foods, possibly due to their non-nutritive components, may contribute to chronic inflammation and gut dysbiosis, which have previously been associated with a pro-inflammatory state [72]. Homeostatic balance and the interplay of immune and metabolic responses are disrupted, which can lead to chronic metabolic inflammation [73]. A diet high in salt or sugar contributes to the infiltration of immune cells and the subsequent release of pro-inflammatory cytokines in tissues [74]. This inflammatory reaction can, for example, prevent glucose uptake and alter lipid metabolism [75]. These metabolic changes can easily contribute to glucose intolerance, excessive weight gain, metabolic inflexibility and ultimately diabetes and cardiovascular disease in susceptible individuals [76, 77]. For example, insulin resistance often results from prolonged exposure to inflammatory biomarkers, which often culminate in the development of diabetes [78]. In addition, low-grade inflammation is associated with atheroprogression and may promote the progression of various cancers through increased cell proliferation, decreased apoptosis and increased angiogenesis and metastasis [79–82].

The present study provides an overview of the effects of different types of caloric diets on biochemical, molecular and behavioral changes and the associated consequences, but may be limited by the lack of some information. Some studies examined only female or male animals, and the articles did not consider sex-specific effects. Although some studies have examined animals at different ages and even the effects on the offspring of mothers exposed to a high-calorie diet, no protocols have been developed that reverse the diet, which is an important limitation. Future studies aimed at filling these gaps are important.

## Conclusion

Our review suggests that a high-calorie diet induces several changes at the peripheral and central nervous system level, leading to neuronal, functional and structural adaptations that may contribute to behavioral changes in the short and long term. These effects could be related to changes in the gut microbiota, glucose and insulin metabolism, and alterations in bile acid signaling pathways. Finally, certain aspects remain unexplored, such as the reversibility of damage caused by high-calorie diets. This underscores the need for future research aimed at mitigating the harmful effects of such diets on the brain. Additionally, studies investigating the different types and sources of high-calorie diets across various species could be crucial for translating these findings to humans.

## Supplementary Information

Below is the link to the electronic supplementary material.

ESM 1 (DOCX 31.3 KB)

## Data Availability

No datasets were generated or analysed during the current study.

## References

[1] Gulyaeva NV (2017) Molecular mechanisms of neuroplasticity: an expanding universe. Biochemistry 82(3):237–242. 10.1134/S0006297917030020 28320264 10.1134/S0006297917030014

[2] Phillips C (2017) Lifestyle modulators of neuroplasticity: how physical activity, mental engagement, and diet promote cognitive health during aging. Neural Plast. 10.1155/2017/3589271 28695017 10.1155/2017/3589271 PMC5485368

[3] Tomasi D, Wang GJ, Volkow ND (2013) Energetic cost of brain functional connectivity. Proc Natl Acad Sci U S A 110(33):13642–13647. 10.1073/pnas.1304896110 23898179 10.1073/pnas.1303346110 PMC3746878

[4] Gómez-Pinilla F (2008) Brain foods: the effects of nutrients on brain function. Nat Rev Neurosci 9(7):568–578. 10.1038/nrn2421 18568016 10.1038/nrn2421 PMC2805706

[5] Muth AK, Park SQ (2021) The impact of dietary macronutrient intake on cognitive function and the brain. Clin Nutr 40(6):3999–4010. 10.1016/j.clnu.2021.04.013 34139473 10.1016/j.clnu.2021.04.043

[6] Diniz CRAF, Crestani AP (2023) The times they are a-changin’: a proposal on how brain flexibility goes beyond the obvious to include the concepts of “upward” and “downward” to neuroplasticity. Mol Psychiatry 28(3):977–992. 10.1038/s41380-022-01677-0 36575306 10.1038/s41380-022-01931-x PMC10005965

[7] Voss P, Thomas ME, Cisneros-Franco JM, de Villers-Sidani É (2017) Dynamic brains and the changing rules of neuroplasticity: implications for learning and recovery. Front Psychol 8:1657. 10.3389/fpsyg.2017.01657 29085312 10.3389/fpsyg.2017.01657 PMC5649212

[8] May A (2011) Experience-dependent structural plasticity in the adult human brain. Trends Cogn Sci 15(10):475–482. 10.1016/j.tics.2011.08.002 21906988 10.1016/j.tics.2011.08.002

[9] Sagi Y, Tavor I, Hofstetter S, Tzur-Moryosef S, Blumenfeld-Katzir T, Assaf Y (2012) Learning in the fast lane: new insights into neuroplasticity. Neuron 73(6):1195–1203. 10.1016/j.neuron.2012.03.001 22445346 10.1016/j.neuron.2012.01.025

[10] El-Sayes J, Harasym D, Turco CV, Locke MB, Nelson AJ (2019) Exercise-induced neuroplasticity: a mechanistic model and prospects for promoting plasticity. Neuroscientist 25(1):65–85. 10.1177/1073858417737928 29683026 10.1177/1073858418771538

[11] Wickham SR, Amarasekara NA, Bartonicek A, Conner TS (2020) The big three health behaviors and mental health and well-being among young adults: a cross-sectional investigation of sleep, exercise, and diet. Front Psychol 11:579205. 10.3389/fpsyg.2020.579205 33362643 10.3389/fpsyg.2020.579205 PMC7758199

[12] Poulose SM, Miller MG, Scott T, Shukitt-Hale B (2017) Nutritional factors affecting adult neurogenesis and cognitive function. Adv Nutr 8(6):804–811. 10.3945/an.117.016020 29141966 10.3945/an.117.016261 PMC5683005

[13] Brookie KL, Best GI, Conner TS (2018) Intake of raw fruits and vegetables is associated with better mental health than intake of processed fruits and vegetables. Front Psychol 9:487. 10.3389/fpsyg.2018.00487 29692750 10.3389/fpsyg.2018.00487 PMC5902672

[14] Molteni R, Barnard RJ, Ying Z, Roberts CK, Gómez-Pinilla F (2002) A high-fat, refined sugar diet reduces hippocampal brain-derived neurotrophic factor, neuronal plasticity, and learning. Neuroscience 112(4):803–814. 10.1016/S0306-4522(02)00104-0 12088740 10.1016/s0306-4522(02)00123-9

[15] Wang C, Li H, Chen C, Yao X, Yang C, Yu Z, Ren J, Ming Y, et al. (2023) High-fat diet consumption induces neurobehavioral abnormalities and neuronal morphological alterations accompanied by excessive microglial activation in the medial prefrontal cortex in adolescent mice. Int J Mol Sci 24(11):9394. 10.3390/ijms24119394 37298345 10.3390/ijms24119394 PMC10253629

[16] Głąbska D, Guzek D, Groele B, Gutkowska K (2020) Fruit and vegetable intake and mental health in adults: a systematic review. Nutrients 12(1):115. 10.3390/nu120100115 31906271 10.3390/nu12010115 PMC7019743

[17] Page MJ, McKenzie JE, Bossuyt PM, Boutron I, Hoffmann TC, Mulrow CD, Shamseer L, Tetzlaff JM, et al. (2021) The PRISMA 2020 statement: an updated guideline for reporting systematic reviews. BMJ 372:n71. 10.1136/bmj.n71 33782057 10.1136/bmj.n71 PMC8005924

[18] Akobeng AK (2005) Principles of evidence based medicine. Arch Dis Child 90(8):837–840. 10.1136/adc.2005.071761 16040884 10.1136/adc.2005.071761 PMC1720507

[19] Hooijmans CR, Rovers MM, de Vries RB, Leenaars M, Ritskes-Hoitinga M, Langendam MW (2014) SYRCLE’s risk of bias tool for animal studies. BMC Med Res Methodol 14:43. 10.1186/1471-2288-14-43 24667063 10.1186/1471-2288-14-43 PMC4230647

[20] Musillo C, Creutzberg KC, Collacchi B, Ajmone-Cat MA, De Simone R, Lepre M, Amrein I, Riva MA, et al. (2023) Bdnf-Nrf-2 crosstalk and emotional behavior are disrupted in a sex-dependent fashion in adolescent mice exposed to maternal stress or maternal obesity. Transl Psychiatry 13(1):399. 10.1038/s41398-023-02701-1 38105264 10.1038/s41398-023-02701-1 PMC10725882

[21] Radford-Smith DE, Probert F, Burnet PWJ, Anthony DC (2022) Modifying the maternal microbiota alters the gut-brain metabolome and prevents emotional dysfunction in the adult offspring of obese dams. Proc Natl Acad Sci U S A 119(9):e2108581119. 10.1073/pnas.2108581119 35197280 10.1073/pnas.2108581119 PMC8892342

[22] Lin C, Lin Y, Luo J, Yu J, Cheng Y, Wu X, Lin Y (2021) Maternal high-fat diet multigenerationally impairs hippocampal synaptic plasticity and memory in male rat offspring. Endocrinology 162(1):bqaa214. 10.1210/endocr/bqaa214 33211807 10.1210/endocr/bqaa214

[23] Chiazza F, Bondi H, Masante I, Ugazio F, Bortolotto V, Canonico PL, Grilli M (2021) Short high fat diet triggers reversible and region specific effects in DCX+ hippocampal immature neurons of adolescent male mice. Sci Rep 11(1):21499. 10.1038/s41598-021-00873-x 34728755 10.1038/s41598-021-01059-y PMC8563989

[24] Beilharz JE, Kaakoush NO, Maniam J, Morris MJ (2016) The effect of short-term exposure to energy-matched diets enriched in fat or sugar on memory, gut microbiota and markers of brain inflammation and plasticity. Brain Behav Immun 57:304–313. 10.1016/j.bbi.2016.06.004 27448745 10.1016/j.bbi.2016.07.151

[25] Yao X, Yang C, Wang C, Li H, Zhao J, Kang X, Liu Z, Chen L, et al. (2022) High-fat diet consumption in adolescence induces emotional behavior alterations and hippocampal neurogenesis deficits accompanied by excessive microglial activation. Int J Mol Sci 23(15):8316. 10.3390/ijms23158316 35955450 10.3390/ijms23158316 PMC9368636

[26] Tsai SF, Wu HT, Chen PC, Chen YW, Yu M, Tzeng SF, Wu PH, Chen PS, et al. (2018) Stress aggravates high-fat-diet-induced insulin resistance via a mechanism that involves the amygdala and is associated with changes in neuroplasticity. Neuroendocrinology 107(2):147–157. 10.1159/000485209 29920496 10.1159/000491018

[27] Tsai SF, Wu HT, Chen PC, Chen YW, Yu M, Wang TF, Wu SY, Tzeng SF, et al. (2018) High-fat diet suppresses the astrocytic process arborization and downregulates the glial glutamate transporters in the hippocampus of mice. Brain Res 1700:66–77. 10.1016/j.brainres.2018.05.019 30009766 10.1016/j.brainres.2018.07.017

[28] Li F, Liu BB, Cai M, Li JJ, Lou SJ (2018) Excessive endoplasmic reticulum stress and decreased neuroplasticity-associated proteins in prefrontal cortex of obese rats and the regulatory effects of aerobic exercise. Brain Res Bull 140:52–59. 10.1016/j.brainresbull.2018.03.007 29630996 10.1016/j.brainresbull.2018.04.003

[29] Carey AN, Gildawie KR, Rovnak A, Thangthaeng N, Fisher DR, Shukitt-Hale B (2019) Blueberry supplementation attenuates microglia activation and increases neuroplasticity in mice consuming a high-fat diet. Nutr Neurosci 22(4):253–263. 10.1080/1028415X.2018.1503064 28931353 10.1080/1028415X.2017.1376472

[30] Kao YC, Wei WY, Tsai KJ, Wang LC (2019) High fat diet suppresses peroxisome proliferator-activated receptors and reduces dopaminergic neurons in the substantia nigra. Int J Mol Sci 21(1):207. 10.3390/ijms210100207 31892244 10.3390/ijms21010207 PMC6981702

[31] Yao X, Zhao J, Yuan Y, Wang C, Yu Z, Huang Z, Chen C, Yang C, et al. (2023) Prolonged early exposure to a high-fat diet augments the adverse effects on neurobehavior and hippocampal neuroplasticity: involvement of microglial insulin signaling. Am J Pathol 193(10):1568–1586. 10.1016/j.ajpath.2023.06.003 37356575 10.1016/j.ajpath.2023.06.005

[32] Liu Y, Hu Z, Wang J, Liao Y, Shu L (2023) Puerarin alleviates depressive-like behaviors in high-fat diet-induced diabetic mice via modulating hippocampal GLP-1R/BDNF/TrkB signaling. Nutr Neurosci 26(10):997–1010. 10.1080/1028415X.2022.2119473 36039913 10.1080/1028415X.2022.2112439

[33] Yang C, Yao X, Zhang H, Wang C, Zhao J, Xu D, Xiao Y, Li Q, et al. (2023) Effects of prolonged high-fat diet consumption starting at different ages on behavioral parameters and hippocampal neuroplasticity in male mice. J Integr Neurosci 22(1):16. 10.1142/S0219635223500021 36722241 10.31083/j.jin2201016

[34] Zhuang H, Yao X, Li H, Li Q, Yang C, Wang C, Xu D, Xiao Y, et al. (2022) Long-term high-fat diet consumption by mice throughout adulthood induces neurobehavioral alterations and hippocampal neuronal remodeling accompanied by augmented microglial lipid accumulation. Brain Behav Immun 100:155–171. 10.1016/j.bbi.2022.05.013 34848340 10.1016/j.bbi.2021.11.018

[35] de Paiva IHR, da Silva RS, Mendonça IP, Duarte-Silva E, Botelho de Souza JR, Peixoto CA (2023) Fructooligosaccharide (FOS) and galactooligosaccharide (GOS) improve neuroinflammation and cognition by up-regulating IRS/PI3K/AKT signaling pathway in diet-induced obese mice. J Neuroimmune Pharmacol 18(3):427–447. 10.1007/s11481-023-10664 37382830 10.1007/s11481-023-10069-8

[36] Reichelt AC, Loughman A, Bernard A, Raipuria M, Abbott KN, Dachtler J, Van TTH, Moore RJ (2020) An intermittent hypercaloric diet alters gut microbiota, prefrontal cortical gene expression and social behaviors in rats. Nutr Neurosci 23(8):613–627. 10.1080/1028415X.2019.1692642 30466372 10.1080/1028415X.2018.1537169

[37] Reichelt AC, Gibson GD, Abbott KN, Hare DJ (2019) A high-fat high-sugar diet in adolescent rats impairs social memory and alters chemical markers characteristic of atypical neuroplasticity and parvalbumin interneuron depletion in the medial prefrontal cortex. Food Funct 10(4):1985–1998. 10.1039/C9FO00145H 30900711 10.1039/c8fo02118j

[38] Marissal-Arvy N, Batandier C, Dallennes J, Canini F, Poulet L, Couturier K, Hininger-Favier I, Moisan MP, et al. (2014) Effect of a high-fat–high-fructose diet, stress, and cinnamon on central expression of genes related to immune system, hypothalamic-pituitary-adrenocortical axis function, and cerebral plasticity in rats. Br J Nutr 111(7):1190–1201. 10.1017/S0007114513004302 24252462 10.1017/S0007114513003577

[39] Jena PK, Sheng L, Nguyen M, Di Lucente J, Hu Y, Li Y, Maezawa I, Jin LW, et al. (2020) Dysregulated bile acid receptor-mediated signaling and IL-17A induction are implicated in diet-associated hepatic health and cognitive function. Biomark Res 8(1):59. 10.1186/s40364-020-00220-7 33292701 10.1186/s40364-020-00239-8 PMC7648397

[40] Jena PK, Setayesh T, Sheng L, Di Lucente J, Jin LW, Wan YY (2022) Intestinal microbiota remodeling protects mice from Western diet-induced brain inflammation and cognitive decline. Cells 11(3):504. 10.3390/cells11030504 35159313 10.3390/cells11030504 PMC8834507

[41] Jena PK, Sheng L, Di Lucente J, Jin LW, Maezawa I, Wan YY (2018) Dysregulated bile acid synthesis and dysbiosis are implicated in western diet-induced systemic inflammation, microglial activation, and reduced neuroplasticity. FASEB J 32(5):2866–2877. 10.1096/fj.201700494R 29401580 10.1096/fj.201700984RR PMC5901391

[42] Beilharz JE, Kaakoush NO, Maniam J, Morris MJ (2018) Cafeteria diet and probiotic therapy: cross talk among memory, neuroplasticity, serotonin receptors and gut microbiota in the rat. Mol Psychiatry 23:351–361. 10.1038/mp.2017.38 28289278 10.1038/mp.2017.38

[43] Reichelt AC, Maniam J, Westbrook RF, Morris MJ (2015) Dietary-induced obesity disrupts trace fear conditioning and decreases hippocampal reelin expression. Brain Behav Immun 43:68–75. 10.1016/j.bbi.2014.08.012 25043993 10.1016/j.bbi.2014.07.005

[44] Squizani S, Jantsch J, Rodrigues FDS, Braga MF, Eller S, de Oliveira TF, Silveira AK, Moreira JCF, et al. (2022) Zinc supplementation partially decreases the harmful effects of a cafeteria diet in rats but does not prevent intestinal dysbiosis. Nutrients 14(19):3921. 10.3390/nu14193921 36235574 10.3390/nu14193921 PMC9571896

[45] Spagnuolo MS, Mazzoli A, Nazzaro M, Troise AD, Gatto C, Tonini C, Colardo M, Segatto M, et al. (2023) Long-lasting impact of sugar intake on neurotrophins and neurotransmitters from adolescence to young adulthood in rat frontal cortex. Mol Neurobiol 60(2):1004–1020. 10.1007/s12035-022-02990-4 36394711 10.1007/s12035-022-03115-8 PMC9849314

[46] Reichelt AC, Loughman A, Bernard A, Raipuria M, Abbott KN, Dachtler J, Van TT, Moore RJ (2020) An intermittent hypercaloric diet alters gut microbiota, prefrontal cortical gene expression and social behaviours in rats. Nutr Neurosci 23(8):613–27. 10.1080/1028415X.2018.1537169 30466372 10.1080/1028415X.2018.1537169

[47] Beilharz JE, Kaakoush NO, Maniam J, Morris MJ (2018) Cafeteria diet and probiotic therapy: cross talk among memory, neuroplasticity, serotonin receptors, and gut microbiota in the rat. Mol Psychiatry 23(2):351–361. 10.1038/mp.2017.125 28289278 10.1038/mp.2017.38

[48] Carey AN, Gildawie KR, Rovnak A, Thangthaeng N, Fisher DR, Shukitt-Hale B (2019) Blueberry supplementation attenuates microglia activation and increases neuroplasticity in mice consuming a high-fat diet. Nutr Neurosci 22(4):253–63. 10.1080/1028415X.2017.1376472 28931353 10.1080/1028415X.2017.1376472

[49] Koehler K, Drenowatz C (2019) Integrated role of nutrition and physical activity for lifelong health. Nutrients 11(7):1437. 10.3390/nu11071437 31247924 10.3390/nu11071437 PMC6682932

[50] Clemente-Suárez VJ, Beltrán-Velasco AI, Redondo-Flórez L, Martín-Rodríguez A, Tornero-Aguilera JF (2023) Global impacts of Western diet and its effects on metabolism and health: a narrative review. Nutrients 15(12):2749. 10.3390/nu15122749 37375654 10.3390/nu15122749 PMC10302286

[51] Blumenthal DM, Gold MS (2010) Neurobiology of food addiction. Curr Opin Clin Nutr Metab Care 13(4):359–365. 10.1097/MCO.0b013e32833fdac8 20495452 10.1097/MCO.0b013e32833ad4d4

[52] Corwin RL, Hajnal A (2005) Too much of a good thing: Neurobiology of non-homeostatic eating and drug abuse. Physiol Behav 86(1–2):5–8. 10.1016/j.physbeh.2005.06.028 16081112 10.1016/j.physbeh.2005.06.021 PMC1769469

[53] Forde CG, Mars M, de Graaf K (2020) Ultra-processing or oral processing? A role for energy density and eating rate in moderating energy intake from processed foods. Curr Dev Nutr 4(3):nzaa019. 10.1093/cdn/nzaa019 32110771 10.1093/cdn/nzaa019 PMC7042610

[54] Marx W, Lane M, Hockey M, Aslam H, Berk M, Walder K, Borsini A, Firth J, et al. (2021) Diet and depression: exploring the biological mechanisms of action. Mol Psychiatry 26(1):134–150. 10.1038/s41380-020-00907-6 33144709 10.1038/s41380-020-00925-x

[55] Small DM, DiFeliceantonio AG (2019) Processed foods and food reward. Science 363(6425):346–347. 10.1126/science.aaw2157 30679360 10.1126/science.aav0556

[56] Damiani F, Cornuti S, Tognini P (2023) The gut-brain connection: exploring the influence of the gut microbiota on neuroplasticity and neurodevelopmental disorders. Neuropharmacology 231:109491. 10.1016/j.neuropharm.2022.109491 36924923 10.1016/j.neuropharm.2023.109491

[57] Murciano-Brea J, Garcia-Montes M, Geuna S, Herrera-Rincon C (2021) Gut microbiota and neuroplasticity. Cells 10(8):2084. 10.3390/cells10082084 34440854 10.3390/cells10082084 PMC8392499

[58] Murphy T, Dias GP, Thuret S (2014) Effects of diet on brain plasticity in animal and human studies: mind the gap. Neural Plast 2014:563160. 10.1155/2014/563160 24900924 10.1155/2014/563160 PMC4037119

[59] Haley MJ, Krishnan S, Burrows D, de Hoog L, Thakrar J, Schiessl I, Allan SM, Lawrence CB (2019) Acute high-fat feeding leads to disruptions in glucose homeostasis and worsens stroke outcome. J Cereb Blood Flow Metab 39(6):1026–1037. 10.1177/0271678X18816453 29171775 10.1177/0271678X17744718 PMC6545621

[60] Antunes MM, Godoy G, Masi LN, Curi R, Barbosa Bazotte R (2022) Prefrontal cortex and hippocampus inflammation in mice fed high-carbohydrate or high-fat diets. J Med Food 25(1):110–113. 10.1089/jmf.2021.0106 34495750 10.1089/jmf.2021.0026

[61] Kanoski SE, Davidson TL (2011) Western diet consumption and cognitive impairment: links to hippocampal dysfunction and obesity. Physiol Behav 103(1):59–68. 10.1016/j.physbeh.2011.01.005 21167850 10.1016/j.physbeh.2010.12.003 PMC3056912

[62] Miller AA, Spencer SJ (2014) Obesity and neuroinflammation: a pathway to cognitive impairment. Brain Behav Immun 42:10–21. 10.1016/j.bbi.2014.06.011 24727365 10.1016/j.bbi.2014.04.001

[63] Beilharz JE, Maniam J, Morris MJ (2015) Diet-induced cognitive deficits: the role of fat and sugar, potential mechanisms and nutritional interventions. Nutrients 7(8):6719–6738. 10.3390/nu7085270 26274972 10.3390/nu7085307 PMC4555146

[64] Sa M, Park MG, Lee CJ (2022) Role of hypothalamic reactive astrocytes in diet-induced obesity. Mol Cells 45(2):65–75. 10.14348/molcells.2022.0035 35236781 10.14348/molcells.2022.2044 PMC8907000

[65] Buckman LB, Thompson MM, Lippert RN, Blackwell TS, Yull FE, Ellacott KL (2014) Evidence for a novel functional role of astrocytes in the acute homeostatic response to high-fat diet intake in mice. Mol Metab 4(1):58–63. 10.1016/j.molmet.2014.10.006 25685690 10.1016/j.molmet.2014.10.001 PMC4314532

[66] Rabot S, Membrez M, Bruneau A, Gérard P, Harach T, Moser M, Raymond F, Mansourian R, et al. (2010) Germ-free C57BL/6J mice are resistant to high-fat-diet-induced insulin resistance and have altered cholesterol metabolism. FASEB J 24(12):4948–4959. 10.1096/fj.10-164452 20724524 10.1096/fj.10-164921

[67] Bolte LA, Vich Vila A, Imhann F, Collij V, Gacesa R, Peters V, Wijmenga C, Kurilshikov A, et al. (2021) Long-term dietary patterns are associated with pro-inflammatory and anti-inflammatory features of the gut microbiome. Gut 70(7):1287–1298. 10.1136/gutjnl-2020-321543 33811041 10.1136/gutjnl-2020-322670 PMC8223641

[68] Zhang X, Monnoye M, Mariadassou M, Beguet-Crespel F, Lapaque N, Heberden C, Douard V (2021) Glucose but not fructose alters the intestinal paracellular permeability in association with gut inflammation and dysbiosis in mice. Front Immunol 12:742584. 10.3389/fimmu.2021.742584 35024040 10.3389/fimmu.2021.742584 PMC8744209

[69] Yeomans MR, Armitage R, Atkinson R, Francis H, Stevenson RJ (2023) Habitual intake of fat and sugar is associated with poorer memory and greater impulsivity in humans. PLoS ONE 18(8):e0290308. 10.1371/journal.pone.0290308 37616232 10.1371/journal.pone.0290308 PMC10449134

[70] Feng Z, Fang C, Ma Y, Chang J (2024) Obesity-induced blood-brain barrier dysfunction: phenotypes and mechanisms. J Neuroinflammation 21(1):110. 10.1186/s12974-024-02740-4 38678254 10.1186/s12974-024-03104-9 PMC11056074

[71] Sánchez-Tapia M, Miller AW, Granados-Portillo O, Tovar AR, Torres N (2020) The development of metabolic endotoxemia is dependent on the type of sweetener and the presence of saturated fat in the diet. Gut Microbes 12(1):1801301. 10.1080/19490976.2020.1801301 32804018 10.1080/19490976.2020.1801301 PMC7524302

[72] Heiman ML, Greenway FL (2016) A healthy gastrointestinal microbiome is dependent on dietary diversity. Mol Metab 5(5):317–320. 10.1016/j.molmet.2016.02.005 27110483 10.1016/j.molmet.2016.02.005 PMC4837298

[73] Asensi MT, Napoletano A, Sofi F, Dinu M (2023) Low-grade inflammation and ultra-processed foods consumption: a review. Nutrients 15(6):1546. 10.3390/nu15061546 36986276 10.3390/nu15061546 PMC10058108

[74] Calder PC, Ahluwalia N, Albers R, Bosco N, Bourdet-Sicard R, Haller D, Holgate ST, Jönsson LS, et al. (2013) A consideration of biomarkers to be used for evaluation of inflammation in human nutritional studies. Br J Nutr 109(1):S1-34. 10.1017/S0007114512005119 23343744 10.1017/S0007114512005119

[75] Hotamisligil GS (2006) Inflammation and metabolic disorders. Nature 444(7121):860–867. 10.1038/nature05485 17167474 10.1038/nature05485

[76] Blüher M (2019) Obesity: global epidemiology and pathogenesis. Nat Rev Endocrinol 15(5):288–298. 10.1038/s41574-019-0176-8 30814686 10.1038/s41574-019-0176-8

[77] Pereira MA (2014) Sugar-sweetened and artificially-sweetened beverages in relation to obesity risk. Adv Nutr 5(6):797–808. 10.3945/an.114.007062 25398745 10.3945/an.114.007062 PMC4224219

[78] Liu C, Xiu F, Li Q, Wang Y, Li Q, Hua M (2016) Adiponectin, TNF-α and inflammatory cytokines and risk of type 2 diabetes: a systematic review and meta-analysis. Cytokine 86:100–109. 10.1016/j.cyto.2016.06.028 27498215 10.1016/j.cyto.2016.06.028

[79] Lawler PR, Bhatt DL, Godoy LC, Lüscher TF, Bonow RO, Verma S, Ridker PM (2021) Targeting cardiovascular inflammation: next steps in clinical translation. Eur Heart J 42(1):113–131. 10.1093/eurheartj/ehaa099 32176778 10.1093/eurheartj/ehaa099

[80] Fouad YA, Aanei C (2017) Revisiting the hallmarks of cancer. Am J Cancer Res 7(5):1016–1036 28560055 PMC5446472

[81] Goncalves MD, Hopkins BD, Cantley LC (2019) Dietary fat and sugar in promoting cancer development and progression. Annu Rev Cancer Biol 3:255–273. 10.1146/annurev-cancerbio-030518-055855

[82] Goncalves MD, Lu C, Tutnauer J, Hartman TE, Hwang S, Murphy CJ, Pauli C, Morris R, et al. (2019) High-fructose corn syrup enhances intestinal tumor growth in mice. Science 363(6433):1345–1349. 10.1126/science.aat8515 30898933 10.1126/science.aat8515 PMC6487857

